# Impact of preoperative skeletal muscle mass on the healing time of postoperative pancreatic fistula after pancreaticoduodenectomy

**DOI:** 10.1007/s00423-026-04029-y

**Published:** 2026-05-08

**Authors:** Takuro Takeuchi, Yoshito Tomimaru, Hirofumi Akita, Yosuke Mukai, Kazuki Sasaki, Shinichiro Hasegawa, Daisaku Yamada, Takehiro Noda, Yuichiro Doki, Hidetoshi Eguchi

**Affiliations:** Department of Gastroenterological Surgery, Graduate School of Medicine, The University of Osaka, 2-2-E2, Yamadaoka,, Suita, Osaka 565-0871 Japan

**Keywords:** Pancreatic fistula, Pancreaticoduodenectomy, Skeletal muscle, SMI

## Abstract

**Background:**

Postoperative pancreatic fistula (POPF) is a serious complication following pancreaticoduodenectomy (PD). While numerous studies have investigated POPF incidence and risk factors, few have focused on POPF healing time (POPF-HT). Skeletal muscle mass has been increasingly recognized as an endocrine organ involved in interorgan communication. The aim of this study was to evaluate how skeletal muscle mass influenced the healing time of POPF after PD.

**Methods:**

This investigation included patients who developed POPF of grade B–C after PD with pancreaticojejunostomy during the study period. POPF-HT was defined as the duration from the date of PD to the removal of all drains placed for POPF treatment. Skeletal muscle mass was assessed based on preoperative computed tomography images. Clinical factors associated with POPF-HT were analyzed, including skeletal muscle index (SMI).

**Results:**

The mean POPF-HT was 40 ± 15 days (median: 37 days; range: 21–105 days). Compared to those with high SMI, patients with low SMI had a significantly longer POPF-HT (46 ± 18 days vs. 37 ± 11 days, *p* = 0.0101). In univariate analysis, low SMI was significantly associated with prolonged POPF-HT (hazard ratio: 0.5655, 95% confidence interval: 0.3687–0.8673, *p* = 0.0090). Kaplan–Meier curves confirmed that patients with low SMI exhibited significantly delayed cumulative POPF healing (*p* = 0.0065).

**Conclusion:**

Decreased skeletal muscle mass is significantly associated with prolonged POPF-HT after PD. Therefore, preoperative SMI may serve as a potentially modifiable factor associated with POPF-HT.

## Introduction

 Pancreaticoduodenectomy (PD) is a standard surgical treatment for benign and malignant diseases of the pancreatic head and uncinate process. While advances in surgical techniques and perioperative management have reduced perioperative mortality, postoperative pancreatic fistula (POPF) remains a significant complication, with reported incidence rates ranging from 20 to 50% [1–3]. POPF development can lead to serious complications—such as intra-abdominal abscess, pseudoaneurysm, and postoperative hemorrhage—potentially resulting in prolonged postoperative hospital stay, higher medical costs, and increased mortality. Therefore, POPF risk prediction has become an important clinical issue, and numerous studies have been conducted to identify factors associated with POPF development. Reported risk factors have included pancreatic texture, main pancreatic duct diameter, body mass index (BMI), sex, operative time, and intraoperative blood loss [2, 4–6]. Moreover, once POPF occurs, it is essential to be able to select an appropriate treatment strategy, and to estimate the duration required for its resolution—a parameter termed POPF healing time (POPF-HT). We previously investigated the time required for healing of POPF after distal pancreatectomy, but few studies have focused on POPF-HT after PD [7, 8].

In recent years, skeletal muscle has attracted increasing attention not only for its mechanical and locomotive roles, but also due to its function as an endocrine organ. A growing body of evidence demonstrates that skeletal muscle secretes various myokines that influence distant organs and systemic physiological processes [9, 10]. Of particular interest, some emerging evidence links skeletal muscle to wound healing. Several experimental studies have indicated that muscle-derived factors can modulate tissue regeneration, promote angiogenesis, and accelerate epithelial and stromal repair. Thus, it is reasonable to hypothesize that skeletal muscle mass may influence the time required for POPF resolution. No reliable predictive factor has yet been established for POPF-HT. If skeletal muscle mass is found to be associated with the POPF-HT, its measurement may allow for better perioperative risk stratification, and enable clinicians to more accurately estimate the expected recovery course.

In the present study, we aimed to investigate the relationship between preoperative skeletal muscle mass and the healing time of POPF following PD. A main goal was to identify a clinically meaningful predictor of POPF resolution.

## Patients and methods

Between January 2010 and December 2024, a total of 602 consecutive patients underwent PD at the Department of Gastroenterological Surgery, The University of Osaka Hospital. Among these patients, 121 developed POPF classified as grade B or C, according to the criteria of the International Study Group on Pancreatic Surgery (ISGPS) [1]. Of these 121 patients, 19 were excluded from this analysis for the following reasons: underwent PD with pancreaticogastrostomy (*n* = 10); had prolonged drain placement due to perioperative complications not associated with POPF, such as chylous ascites (*n* = 5); underwent PD with hepatic resection (*n* = 3); and could not undergo skeletal muscle index (SMI) assessment due to spinal metallic implants causing imaging artifacts (*n* = 1). Consequently, 102 patients were included in the analysis of clinical factors associated with POPF-HT. Informed consent for the use of clinical data was obtained from all patients through a comprehensive consent process at the time of admission. In addition, an opt-out method was employed to provide patients with the opportunity to decline participation in this specific study, in accordance with the protocol approved by the Institutional Ethics Review Committee of The University of Osaka Hospital (Approval No. 25167).

In this study, POPF-HT was defined as the period from the date of PD to the date when the POPF was considered healed, i.e., the date of removal of all intra-abdominal drainage tubes placed for POPF management [11]. SMI was evaluated using axial plane computed tomography (CT) images at the level of the third lumbar vertebra (L3) [12, 13]. Skeletal muscle area—including the psoas major, erector spinae, quadratus lumborum, transversus abdominis, internal and external oblique muscles, and rectus abdominis—was semi-automatically measured using SYNAPSE VINCENT (Fujifilm Medical Co., Ltd.). Muscle tissue was identified within a Hounsfield unit range of − 29 to + 150. SMI was calculated as the skeletal muscle area (cm²) divided by the height squared (m²), and was expressed in cm²/m² [14–16]. Low SMI was defined according to the 2021 revised guidelines of the Japan Society of Hepatology, as SMI < 42 cm²/m² for males, and SMI < 38 cm²/m² for females [17–19]. Visceral fat area (VFA) was evaluated using the same axial CT image at the level of the third lumbar vertebra (L3). Adipose tissue was identified using a predefined Hounsfield unit range of − 190 to − 30 [20]. The intra-abdominal adipose tissue area was semi-automatically segmented using SYNAPSE VINCENT, and only fat located within the abdominal muscular wall was included in the measurement. On contrast-enhanced CT images, the main pancreatic duct diameter was measured at the site of pancreatic transection, and pancreatic thickness was measured above the portal vein. Comorbidities were defined as any pre-existing diseases potentially considered as surgical risk factors, including diabetes mellitus, cardiovascular disease, chronic lung disease, chronic liver disease, and renal failure. The Fistula Risk Score (FRS) was calculated for each patient according to the method proposed by Callery et al. [2]. The FRS incorporates four intraoperative variables: pancreatic texture (soft vs. firm), pathology (pancreatic ductal adenocarcinoma vs. other), main pancreatic duct diameter, and intraoperative blood loss. The total score ranges from 0 to 10, with higher scores indicating a greater risk of clinically relevant POPF.

For all included patients, PD was performed as subtotal stomach-preserving pancreaticoduodenectomy. Reconstruction was achieved using a modified Child method, including pancreaticojejunostomy, hepaticojejunostomy, and gastrojejunostomy with Braun’s anastomosis. Before surgery completion, two closed intra-abdominal drains were placed near the pancreaticojejunostomy, and one was placed near the hepaticojejunostomy. POPF was diagnosed according to the ISGPS classification [1]. All patients received standardized postoperative management, including POPF treatment. Drain fluid amylase concentrations were measured on postoperative days 1 and 3. For patients who developed POPF, intra-abdominal drains were assessed via fistulography and clinical evaluation every 1–2 weeks. Drains were removed once fistulography confirmed the resolution of the fluid collection and the patient was clinically asymptomatic, regardless of drain fluid amylase levels or appearance. CT was utilized additionally if the clinical presentation suggested that the fistula or associated complications, such as secondary infection or undrained collections, extended beyond the findings visible on fistulography. In patients with an amylase concentration exceeding 5,000 U/L and a high volume of drainage, octreotide was administered during the first postoperative week to facilitate closure. To minimize measurement variability and clinician bias, all management strategies, including the specific timing of drain removal, were standardized through daily multidisciplinary conferences involving all staff surgeons of the Hepato-Biliary-Pancreatic surgery team.

Continuous variables were expressed as mean ± standard deviation, and categorical variables as absolute numbers. Intergroup comparisons were performed using the Mann-Whitney U test, chi-squared test, or Fisher’s exact test, as appropriate. POPF-HT was estimated using the Kaplan-Meier method, and cumulative healing rates were compared using the log-rank test. To identify clinical factors associated with POPF-HT, univariate analysis was performed using Cox proportional hazards models, with POPF healing as the event of interest. SMI was dichotomized based on established cut-offs. Statistical analyses were performed using JMP^®^ (SAS Inc., Cary, NC), and *p* values of < 0.05 were considered statistically significant.

## Results

**Fig. 1:**
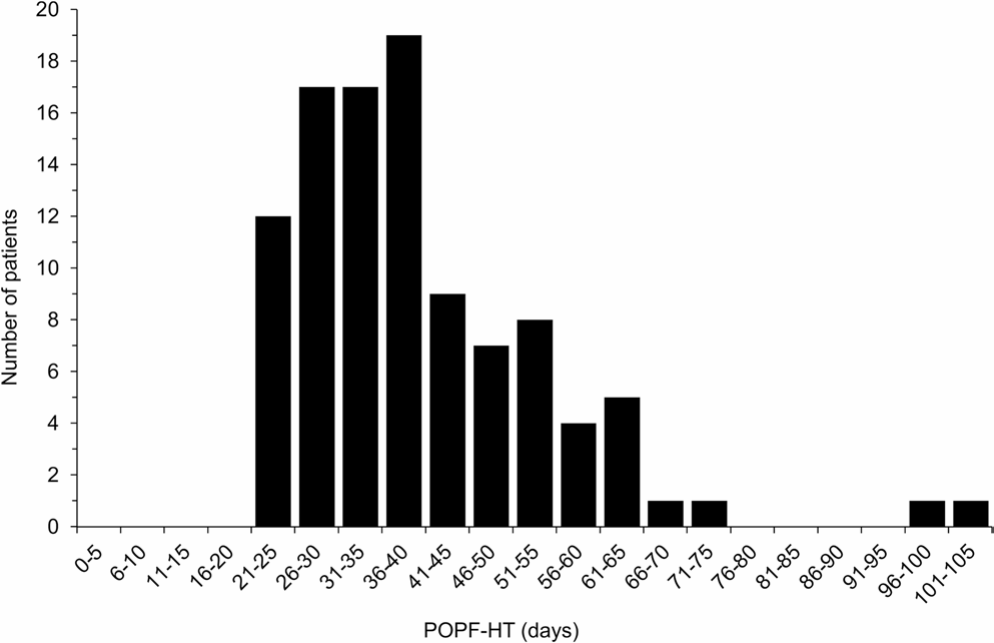
Distribution of the included patients, stratified according to POPF-HT range. Each bar indicates the number of patients in each healing time category. POPF-HT, postoperative pancreatic fistula healing time

Of the 102 included patients, 101 had grade B POPF, and 1 had grade C POPF. Table 1 summarizes the patients’ clinical and surgical characteristics. Figure 1 shows the distribution of POPF-HT for each patient. The mean POPF-HT was 40 ± 15 days (median: 37 days; range: 21–105 days). The mean SMI was 43.8 ± 7.9 cm^2^/m^2^ (median: 44.1 cm^2^/m^2^; range: 29.3–63.3 cm^2^/m^2^), and the patients were divided into two groups, based on the sarcopenia criteria proposed by the Japan Society of Hepatology. Table 1 presents the comparison between these two groups, which revealed significant differences in sex, height, weight, body mass index (BMI), underlying disease, neoadjuvant chemotherapy, and VFA. Compared to patients with high SMI, those with low SMI had a significantly longer healing time (46 ± 18 days vs. 37 ± 11 days, *p* = 0.0101). Regarding other clinical outcomes, postoperative hemorrhage occurred in three patients (8.8%) with low SMI and seven patients (10.3%) with high SMI (*p* = 0.8139). Notably, drain reinsertion was required significantly more often in the low SMI group than in the high SMI group (29.4% vs. 7.4%, *p* = 0.0030). Additionally, the length of postoperative hospital stay was significantly longer in the low SMI group compared to the high SMI group (61 ± 20 days vs. 52 ± 15 days, *p* = 0.0241).

**Table 1:** Perioperative characteristics of all cases, and of the low and high SMI groups

Characteristic		All cases (*n* = 102)	Low SMI (*n* = 34)	High SMI (*n* = 68)	*p* value Low vs. high SMI groups
Preoperative factors					
Age, years		70 ± 10	71 ± 9	69 ± 10	0.1445
Sex, male/female		78/24	17/17	61/7	< 0.0001
Height, cm		163.7 ± 9.2	159.9 ± 9.3	165.6 ± 8.6	0.0040
Weight, kg		62.2 ± 11.1	53.9 ± 8.4	66.4 ± 10.0	< 0.0001
BMI, kg/m^2^		23.2 ± 3.2	21.1 ± 2.8	24.2 ± 2.9	< 0.0001
Disease, pancreatic cancer/others		26/76	14/20	12/56	0.0102
Neoadjuvant therapy, no/yes		81/21	20/14	61/7	0.0003
Comorbidities, no/yes		21/81	7/27	14/54	1.0000
White blood cells, /µL		5761 ± 2458	5544 ± 2016	5796 ± 2703	0.5978
Neutrophils, /µL		3592 ± 2320	3424 ± 1705	3676 ± 2581	0.5585
Lymphocytes, /µL		1523 ± 602	1535 ± 524	1517 ± 642	0.8844
Platelets, 10^4^/µL		22.7 ± 11.8	24.2 ± 15.9	22.0 ± 9.3	0.4664
Albumin, g/dL		3.9 ± 0.4	3.8 ± 0.42	3.9 ± 0.40	0.4602
Total cholesterol, mg/dL		189 ± 34	182 ± 36	193 ± 33	0.1630
CRP, mg/dL		0.74 ± 1.8	0.60 ± 1.3	0.81 ± 2.0	0.5009
PNI		38.8 ± 4.1	38.4 ± 4.2	39.1 ± 4.0	0.4657
NLR		2.9 ± 3.5	2.4 ± 1.2	3.2 ± 4.2	0.1643
MPD diameter, mm		2.5 ± 2.1	2.3 ± 2.2	2.6 ± 2.0	0.4558
Pancreas thickness, mm		13.5 ± 4.2	13.1 ± 5.4	13.7 ± 3.5	0.5993
SMI, cm^2^/m^2^		43.8 ± 8.0	35.2 ± 3.5	48.2 ± 5.6	< 0.0001
VFA, cm^2^/m^2^		152.5 ± 77.8	110.5 ± 57.8	172.1 ± 71.8	< 0.0001
Intraoperative factors					
Surgical approach, open/laparoscopic		95/7	32/2	63/5	0.7818
Operation time, min		520 ± 100	540 ± 125	510 ± 84	0.2086
Intraoperative blood loss, mL		715 ± 477	688 ± 455	729 ± 491	0.6766
Pancreatic texture, soft/firm		71/31	20/14	51/17	0.0940
FRS		6.5 ± 2.1	6.3 ± 2.1	6.6 ± 2.1	0.5314
Postoperative factors					
POPF, Grade B/Grade C		101/1	33/1	68/0	0.1553
Amylase concentration in the drainage fluid on POD3, U/L		10,727 ± 30,686	17,330 ± 50,279	7426 ± 11,880	0.2650
Octreotide, no/yes		19/83	8/26	11/57	0.3686
Postoperative hemorrhage, no/yes		92/10	31/3	61/7	0.8139
Drain reinsertion, no/yes		87/15	24/10	63/5	0.0030
Postoperative hospital stay, days		55 ± 17	61 ± 20	52 ± 15	0.0241

Data are expressed as the mean ± standard deviation for continuous variables, or the number of patients for categorical variables

Abbreviations: BMI, body mass index; CRP, C-reactive protein; FRS, fistula risk score; MPD, main pancreatic duct; NLR, neutrophil-to-lymphocyte ratio; PNI, prognostic nutritional index; POD, postoperative day; POPF, postoperative pancreatic fistula; SMI, skeletal muscle index; VFA, visceral fat area

To further identify factors potentially associated with POPF-HT, we performed univariate analysis using preoperatively known factors, including SMI (Table 2). Univariate analysis revealed that SMI was significantly associated with POPF-HT [hazard ratio (HR): 0.5655, 95% confidence interval (95% CI), 0.3687–0.8673, *p* = 0.0090].

**Table 2:** Univariate analysis to identify factors associated with POPF-HT

Factors	Number	Univariate		
		HR	95% CI	*P* value
Age, years, ≤71 vs. >71	52/50	1.4288	0.9640–2.1177	0.0755
Sex, male vs. female	78/24	1.0590	0.5953–1.4980	0.8079
Height, cm, ≤164 vs. >164	51/51	1.0729	0.7217–1.5949	0.7281
Weight, kg, ≤63 vs. >63	51/51	0.9571	0.6473–1.4153	0.8262
BMI, kg/m^2^, ≤23 vs. >23	51/51	0.8984	0.6064–1.3312	0.5933
Disease, pancreatic cancer vs. others	26/76	1.0108	0.6457–1.5824	0.9625
Neoadjuvant therapy, no vs. yes	81/21	1.0812	0.6664–1.7541	0.7519
Comorbidities, no vs. yes	21/81	1.3080	0.8033–2.1297	0.2804
White blood cells, /μL, ≤5200 vs. >5200	51/51	0.8110	0.5472–1.2019	0.2966
Neutrophils, /μL, ≤3127 vs. >3127	51/51	0.9346	0.6320–1.3820	0.7348
Lymphocytes, /μL, ≤1443 vs. >1443	51/51	0.8854	0.5975–1.3121	0.5442
Platelets, 10^4^/μL, ≤20.8 vs. >20.8	51/51	0.7725	0.5217–1.1439	0.1975
Albumin, g/dL, ≤3.9 vs. >3.9	53/49	0.7158	0.4805–1.0665	0.1003
CRP, mg/dL, ≤0.075 vs. >0.075	51/51	1.1229	0.7514–1.6782	0.5717
Total cholesterol, mg/dL, ≤191 vs. >191	52/50	0.8267	0.5568–1.2276	0.3455
PNI, ≤39 vs. >39	51/51	0.7915	0.5317–1.1783	0.2494
NLR, ≤2.1 vs. >2.1	50/52	1.2706	0.8521–1.8948	0.2401
MPD diameter, mm, ≤1.7 vs. >1.7	52/50	1.1714	0.7903–1.7364	0.4307
Pancreas thickness, mm, ≤13.0 vs. >13.0	54/48	1.0535	0.7080–1.5676	0.7971
SMI, cm^2^/m^2^, <42 vs. ≥42 for males, <38 vs. ≥38 for females	34/68	0.5655	0.3687–0.8673	0.0090
VFA, cm^2^/m^2^, <156.6 vs. ≥156.6 for males, <108.9 vs. ≥108.9 for females	51/51	1.1839	0.7976–1.7574	0.4021

Abbreviations: POPF-HT, postoperative pancreatic fistula healing time; BMI, body mass index; 95% CI, 95% confidence interval; HR, hazard ratio; CRP, C-reactive protein; MPD, main pancreatic duct; NLR, neutrophil-to-lymphocyte ratio; PNI, prognostic nutritional index; POD, postoperative day; SMI, skeletal muscle index; VFA, visceral fat area

The other analyzed factors—including factors that significantly differed between the low and high SMI groups, such as sex, height, weight, BMI, disease, neoadjuvant therapy, and VFA—were not significantly associated with POPF-HT. To address potential confounding by sex, we performed sex-stratified analyses. In the male cohort (*n* = 78), univariate analysis showed that low SMI (HR: 0.4452, 95% CI: 0.2485–0.7977, *p* = 0.0065) was significantly associated with prolonged POPF-HT. Multivariate analysis confirmed that low SMI remained an independent predictor in males (HR: 0.4965, 95% CI: 0.2705–0.9112, *p* = 0.0238). In the female cohort (*n* = 24), no significant association was observed (HR: 0.8353, 95% CI: 0.3241–2.1530, *p* = 0.7095).

**Fig. 2:**
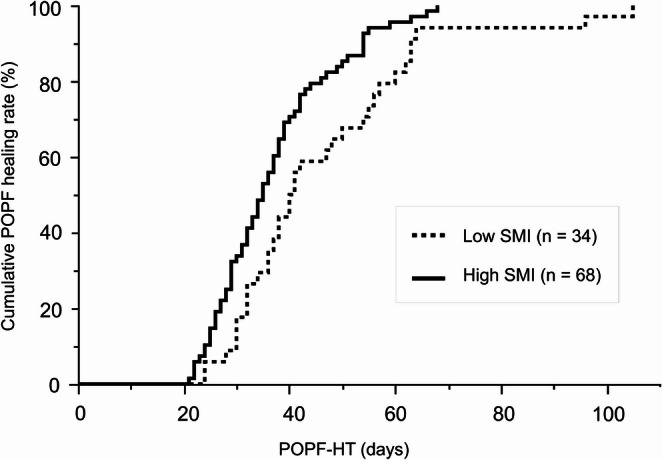
Cumulative POPF healing rate in patients stratified according to SMI. The cumulative POPF healing rate was calculated using the Kaplan-Meier method. The healing time was significantly shorter for patients with high SMI (≥ 42 cm^2^/m^2^ for males, ≥ 38 cm^2^/m^2^ for females), compared to patients with low SMI (< 42 cm^2^/m^2^ for males, < 38 cm^2^/m^2^ for females) (*p* = 0.0065). SMI, skeletal muscle index; POPF, postoperative pancreatic fistula

Based on these findings, Kaplan-Meier analysis was conducted to compare cumulative POPF healing rates in the SMI-defined groups (Fig. 2). This revealed significantly faster cumulative healing in patients with higher SMI, compared to those with low SMI (*p* = 0.0065).

## Discussion

In this study, we analyzed POPF-HT in patients who developed POPF after PD, and investigated the impact of preoperative skeletal muscle mass on this healing. Our analyses revealed that low SMI, which indicates sarcopenia, was significantly associated with prolonged POPF-HT in univariate analysis. These results suggest that preoperative SMI may be a useful predictor of POPF-HT. Although previous studies have reported that skeletal muscle mass is associated with the occurrence of postoperative complications in gastrointestinal and hepatobiliary surgery [21–27], the relationship between muscle mass and healing time has not previously been shown. Regarding the occurrence of POPF itself, previous studies have yielded inconsistent results. A recent meta-analysis including nearly 3,000 patients demonstrated no significant relationship between preoperative low SMI and POPF occurrence [28]. While these reports suggest that skeletal muscle mass may not be a significant risk factor for the POPF development, our findings provide a novel clinical insight: low SMI may be a useful predictor of prolonged recovery once POPF has occurred. Therefore, the present study provides a valuable demonstration of the previously unknown relationship between skeletal muscle mass and POPF-HT. Moreover, low SMI was significantly associated with a higher rate of drain reinsertion and a longer postoperative hospital stay. These objective results suggest that prolonged POPF-HT reflects clinical difficulty in fistula resolution rather than mere variations in institutional drain management. Furthermore, these results are meaningful considering their potential for future development, such as the possibility that increasing skeletal muscle mass may shorten POPF-HT.

Skeletal muscle is not merely a locomotive organ; in recent years, it has been increasingly recognized as an endocrine organ [29, 30]. It exerts systemic effects by communicating with various organs—such as the liver, adipose tissue, pancreas, brain, and immune system—thereby influencing glucose and lipid metabolism, insulin sensitivity, neurocognitive function, and immune homeostasis. The cytokines secreted from skeletal muscle, termed myokines, play essential roles in these systemic effects. Indeed, greater skeletal muscle mass is thought to promote increased myokine production, which could help bring about the above-mentioned effects. For example, Zhao et al. reported that greater skeletal muscle mass was associated with higher irisin secretion—which, in turn, strengthened its beneficial effects on sarcopenia and liver function [31]. In this setting, the present results lead us to hypothesize that patients with low SMI might exhibit altered myokine profiles, potentially influencing the biological processes required for POPF resolution. However, as we did not measure specific myokine levels, this remains a speculative mechanism that warrants further investigation. Beyond myokine-mediated effects, low SMI may reflect poor nutritional status, physical frailty, or sarcopenic obesity. These interrelated factors can impair inflammatory responses and tissue repair. While SMI was an independent predictor in our analysis, these systemic conditions could collectively contribute to delayed POPF healing.

In terms of the clinical application of this study, the results suggest that preoperative SMI may represent a potentially modifiable factor associated with POPF-HT. In turn, shortening POPF-HT after PD could offer three major clinical benefits. First, it may reduce the risk of POPF-induced secondary complications—such as intra-abdominal abscesses and pseudoaneurysms, which can lead to postoperative hemorrhage. Second, it may lower the overall cost of treatment. Third, when patients receive PD to treat malignant diseases, such as pancreatic cancer, shortening the POPF-HT may prevent delays in the initiation of adjuvant chemotherapy, which may be crucial for improving prognosis [32].

The present study has several limitations. First, we did not measure myokine levels in the patients in this study. It would be desirable to directly measure myokine levels, to test the hypothesis that higher skeletal muscle mass leads to increased myokine secretion, thereby shortening POPF-HT. Unfortunately, this was not possible due to the shortage of blood samples from the included patients. Furthermore, sex-stratified analysis revealed that SMI was a robust predictor in male patients but not in the female cohort. This lack of significance in females is likely due to the limited statistical power from the small sample size (*n* = 24). Second, our analysis relied on the use of specific cut-off values for SMI. In this study, we used the criteria established by the Japan Society of Hepatology, which are considered well-suited to the Japanese population but may vary depending on race and institutional measurement standards. Therefore, caution should be used when applying these results in other countries.

In conclusion, low preoperative skeletal muscle mass was associated with prolonged POPF-HT after PD. Assessment of skeletal muscle mass may help identify patients at risk for delayed healing, and preoperative SMI may represent a potentially modifiable factor associated with POPF-HT.

## Data Availability

The datasets generated and/or analyzed during the current study are available from the corresponding author on reasonable request.

## Declarations

### Registry and the registration no. of the study/trial

N/A.

### Animal studies

N/A.
